# Self-Reported Physical Limitations Among U.S. Veterans Compared to Non-Veterans: Findings from NHANES

**DOI:** 10.1093/geroni/igab046.2393

**Published:** 2021-12-17

**Authors:** Ronna Robbins, Monica Serra, Odessa Addison

**Affiliations:** 1 Audie Murphy VA Medical Center, San Antonio, Texas, United States; 2 UT Health San Antonio - Barshop Institute, San Antonio, Texas, United States; 3 University of Maryland School of Medicine, Baltimore, Maryland, United States

## Abstract

Approximately 43% of males over the age of 65 years are Veterans. Veterans may be at elevated risk for functional declines due to barriers to health care access leading to accelerated loss of independence. This compared the prevalence of functional limitations in Veterans and non-Veterans. Data from two National Health and Examination Survey collection periods, administered 2013-2014 and 2015-2016, were used to compare physical functioning data between male Veterans (N=369) and non-Veterans (N=738) matched 1:2 for sex, race, and BMI. Individuals were considered a Veteran if they self-reported having “served in active duty in the U.S. Armed forces.” Pearson’s chi-square tests were used to assess differences in the prevalence of various self-reported functional limitations between groups. Veterans (mean±SEM: age: 64.5±0.54 years; BMI: 30.0±0.3 kg/m2) were disproportionately affected by self-reported functional limitations caused by long-term physical, mental, or emotional illnesses (8% vs. 3%, p<0.004). Twenty-five % of Veterans reported that these limitations kept them from working compared to 18% of non-Veterans (p<0.003). Veterans (38%) were also more likely to report being limited in the amount of work they could perform compared to non-Veterans (27%) (p<0.01). Additionally, Veterans (20%) were more likely to report the use of special healthcare equipment (i.e. cane, wheelchair) than non-Veterans (12%) (p<0.001). These data suggest that Veterans are at greater risk for functional limitations caused by self-reported long-term physical, mental or emotional illness. Therefore, further research is needed to determine if home- and community-based services could prevent further functional decline, ultimately allowing Veterans to maintain independence.

